# Prediction model for risk of multiple organ dysfunction syndrome in wasp sting patients: development and validation

**DOI:** 10.3389/fmed.2026.1834165

**Published:** 2026-06-25

**Authors:** Hongmei Dai, Dongmei Liu, Li Yang, Jiawei Chen, Xiaogang Du

**Affiliations:** 1 Department of Nephrology and Hematology, Yunyang County People’s Hospital, Yunyang County, Chongqing, China; 2 Health Management Center, The First Affiliated Hospital of Chongqing Medical University, Chongqing, China; 3 Metabolism and Immunology Laboratory for Urological Diseases, The First Affiliated Hospital of Chongqing Medical University, Chongqing, China

**Keywords:** associated factors, multiple organ dysfunction syndrome, nomogram, risk prediction model, wasp sting

## Abstract

**Objective:**

Multiple organ dysfunction syndrome (MODS) is a life-threatening complication of wasp stings. This study aimed to identify factors associated with MODS and develop a quantitative risk prediction model.

**Methods:**

A retrospective cohort study included 324 wasp sting patients (January 2018–December 2023), divided into MODS (*n* = 90) and non-MODS (*n* = 234) groups. General characteristics, imaging findings, and laboratory parameters were compared. Composite indicators with clinical interpretability were incorporated into binary logistic regression to identify risk factors. A nomogram-based prediction model was established and evaluated using ROC curves, calibration curves, and decision curves.

**Results:**

Significant differences were observed between groups in age, time from sting to admission, pleural effusion, RALE score, PLT, LYM, MO, FIB, ALB, MPV/PLT, P-LCR, FAR, FPR, CLR, SHR, NLR, PLR, PNR, SIRI, and SII (all *P* < 0.05). After excluding collinear variables (VIF > 5), binary logistic regression identified age, time to admission, pleural effusion, RALE score, MPV/PLT, and SIRI as independent risk factors (OR > 1, all *P* < 0.05). A nomogram prediction model was developed. ROC analysis showed an AUC of 0.828 (95% CI: 0.776–0.880). At the optimal cutoff of 0.368, sensitivity was 66.7%, specificity 87.9%, PPV 68.9%, and NPV 86.8%. Calibration curves showed good agreement, and decision curves demonstrated high net clinical benefit.

**Conclusion:**

MODS occurrence after wasp stings may be associated with RALE score, MPV/PLT, and SIRI levels, while the roles of age, time to admission, and pleural effusion require further validation. The nomogram model provides a useful reference for clinical MODS risk assessment but lacks external validation, requiring confirmation in future large-scale, multicenter, prospective studies.

## Introduction

Stings caused by Hymenoptera are a common form of animal-induced injury worldwide, and among them, wasp stings are particularly dangerous because of their potent venom and high aggressiveness (1). Unlike honeybees, the sting apparatus of wasps is not barbed, so repeated attacks on the same target can be delivered, resulting in the injection of a large amount of venom into the human body. Wasp venom has a complex composition; in addition to biogenic amines and various enzymes, it contains higher levels of specific toxic proteins with stronger cytotoxic, neurotoxic and hemolytic activities. These components more intensely activate the complement system and trigger immune–inflammatory cascades, thereby more readily inducing severe systemic toxic manifestations, and the case-fatality rate is markedly higher than that observed after ordinary honeybee stings (2). Following a wasp sting, venom components quickly enter the systemic circulation and can cause an uncontrollable systemic inflammatory response syndrome (SIRS), which is characterized by widespread activation of inflammatory pathways and cytokine release, in addition to causing prominent local manifestations like erythema, swelling, intense pain, and even frank tissue necrosis at the sting site (3). The most deadly and difficult-to-treat consequence of wasp stings is multiple organ dysfunction syndrome (MODS), which has been shown to have a death rate of over 40% in those who are afflicted (4, 5), highlighting its clinical significance. The clinical syndrome known as MODS is characterized by progressive and possibly reversible or irreversible dysfunction of two or more organ systems that develops over time after a severe systemic insult, such as venom-induced inflammatory and immune dysregulation. If treatment is not received, this can lead to multi-organ failure, following the major insult of wasp venom, dysregulated activation of intrinsic inflammatory, immune and coagulation pathways leads to progressive impairment of the function of two or more organ systems (6, 7). Once MODS develops in a patient with a wasp sting, the risk of death increases sharply, health-care resource utilization becomes substantial, and a heavy burden is imposed on both families and society (8). In order to improve overall prognosis, lower mortality, and maximize the use of medical resources in this vulnerable patient group, early and accurate identification of individuals at high risk for MODS and the timely initiation of appropriate interventions aimed at interrupting or slowing the pathological cascade toward multi-organ failure are crucial.

At present, assessment of disease severity in patients with wasp stings in clinical practice is largely based on empirical judgment and a few isolated clinical or laboratory indicators, such as estimated venom load, the severity of the initial allergic reaction, or serum liver and renal function parameters (9, 10). However, the development of MODS after a wasp sting is a complex process driven by multiple factors and pathways, and any single index is insufficient to capture the dynamic risk comprehensively; this fragmented evaluation strategy has obvious limitations (11). The determinants of MODS in this population have not yet been fully elucidated in clinical studies, and a practical tool that can integrate these key variables and yield an individualized risk probability is still lacking. In addition, the outputs of conventional statistical models are not intuitive for clinicians without a background in biostatistics and are difficult to implement rapidly in fast-paced settings such as emergency departments (12). A nomogram, as a graphical calculation instrument, converts a complex regression equation into an intuitive visual scoring system. With such a tool, clinicians are not required to perform complicated computations; by simply summing the points assigned to each predictor, the individual probability of developing MODS can be rapidly estimated at the bedside. This one-stop integrated and visualized presentation markedly enhances the usability of the model and the efficiency of decision support, and facilitates a shift from experience-based assessment to precise quantitative prediction (13). Consequently, there is an urgent clinical need to construct a comprehensive prediction model for MODS after wasp stings that integrates multidimensional patient indices and remains easy to apply in routine practice.

In this context, a retrospective cohort was established in the present study to analyze factors associated with the occurrence of MODS after wasp stings. On this basis, a visual risk prediction model incorporating baseline clinical characteristics, imaging findings and laboratory parameters was developed and subsequently validated with respect to its reliability and practicality. It is anticipated that this model will become a powerful tool for clinicians—particularly those working in emergency medicine and intensive care—to achieve early and accurate identification of high-risk patients with wasp stings, thereby providing a scientific and effective reference for individualized monitoring and proactive treatment, ultimately reducing the incidence and mortality of MODS related to wasp stings and improving patient outcomes.

## Materials and methods

### Study population

A retrospective cohort study was carried out using a cohort of 324 patients who presented with wasp stings and were consecutively admitted to our hospital over the time span from January 2018 to December 2023, thereby forming a continuous and non-selective series of cases suitable for observational analysis. According to whether MODS developed at any point during the index hospitalization, all enrolled patients were subsequently stratified into two distinct groups: a MODS group (*n* = 90), consisting of individuals who experienced MODS, and a non–multiple organ dysfunction syndrome (NMODS) group (*n* = 234), consisting of those who did not develop this complication. The entire study protocol was reviewed and formally approved by the hospital ethics committee, and all research procedures, including data collection and analysis, were performed in strict adherence to the ethical principles and guidelines set forth in *the Declaration of Helsinki* to protect patient rights and confidentiality.

### Inclusion and exclusion criteria

The inclusion criteria were as follows: (1) a clear history of wasp sting, with wasp sting recorded as the primary admission diagnosis; (2) age ≥ 18 years; (3) absence of MODS at the time of admission; (4) length of hospital stay ≥ 24 h; (5) complete clinical data available.

The exclusion criteria were as follows: (1) pre-existing hepatic, renal, biliary, or hematologic disease, or other chronic disorders with active disease status leading to organ dysfunction; (2) history of treatment with corticosteroids, immunosuppressive agents, or antiallergic drugs within 1 month before the wasp sting; (3) receipt of corticosteroids or antiallergic drugs, mechanical ventilation, or blood purification therapy in another hospital after the wasp sting; (4) continuous clinical deterioration without progression to MODS followed by discharge; (5) stings caused by insects other than wasps.

### General data

For every participant in the cohort, a comprehensive set of baseline general data was systematically collected, including demographic characteristics (sex and age), body mass index (BMI) as an indicator of nutritional and obesity status, vital signs such as respiratory rate and heart rate, systolic and diastolic blood pressure at admission, the anatomical site of the sting, and the intensity of pain as quantified by the visual analog scale (VAS) pain score. The VAS was operationally defined as a straight line of approximately 10 cm in length, either horizontal or vertical, on which both ends were clearly annotated with descriptive terms or facial expression icons indicating the extremes of pain perception; the left endpoint was marked as 0 to represent the complete absence of pain, whereas the right endpoint was labeled as 10 to represent the most severe pain imaginable to the patient. Each patient was instructed to indicate a single point along this line that best reflected their subjective perception of current pain intensity, and the corresponding numerical value derived from the position of this mark was then recorded as the VAS pain score for use in subsequent statistical analyses, with higher scores indicating more severe pain (14). Additional variables included time from sting to hospital admission, number of sting sites, presence of erythema and swelling, pruritus, central necrosis, fever, allergic reactions, respiratory system symptoms, gastrointestinal symptoms, cardiovascular symptoms, urinary system symptoms, and neurological symptoms.

### Chest radiography

Chest radiograph findings obtained within 24 h after admission were collected for all patients. Digital posteroanterior and lateral chest radiographs were acquired in the resting state. A fixed digital X-ray radiography system was used (manufacturer: Shanghai United Imaging Healthcare Co., Ltd., China; model: uDR 770i). To ensure consistent image quality and comparability of radiographic findings across various patients, the imaging parameters of the digital X-ray system were standardized for all chest radiographic examinations included in the study. The focus-to-detector distance was fixed at 180 cm, the tube voltage was continuously maintained within a range of 100–120 kV, and the tube current was controlled by an automatic tube current modulation program. Patients were examined in the standing position and were instructed to hold their breath at full inspiration during exposure. All chest radiographs were independently reviewed and evaluated in a double-blind manner by two senior radiologists who were unaware of the clinical grouping. In addition to recording the presence or absence of pleural effusion, cardiac enlargement, pulmonary congestion, and other radiographic findings, the radiographic assessment of lung edema (RALE) was applied to quantitatively assess the extent and density of consolidation and ground-glass opacities in both lungs. The scoring system was defined as follows: each lung field was divided into upper, middle, and lower zones, yielding a total of six regions. For each region, the extent of involvement by consolidation or ground-glass opacity was scored (1 point: 0–25%; 2 points: 26–50%; 3 points: 51–75%; 4 points: 76–100%), and the density of opacification was scored separately (1 point: ground-glass opacity; 2 points: consolidation). The regional score was calculated as the product of the extent score and the density score. Each patient’s total RALE score was determined by adding up the individual scores allocated to each of the predetermined lung regions. This produced an overall semi-quantitative index of the density and extent of pulmonary opacities, with 48 points being the highest possible theoretical value on this scale (15).

### Complete blood count testing

For hematologic evaluation, complete blood count (CBC) parameters that had been measured within the first 24 h after admission were collected for all patients, allowing characterization of the early systemic inflammatory and hematologic response to wasp envenomation. In the early morning after an overnight fast, approximately 2 mL of venous blood was drawn from the antecubital vein under sterile conditions and immediately transferred into a vacuum collection tube prefilled with dipotassium ethylenediaminetetraacetic acid (EDTA-K_2_), which served as the anticoagulant to prevent clot formation and preserve cellular elements. Following collection, each tube was gently inverted by hand 5–8 times to ensure thorough mixing of the whole blood with the EDTA-K_2_ solution, thereby promoting adequate anticoagulation and minimizing the likelihood of microclot formation that could interfere with accurate cell counting. All CBC samples were processed within 2 h of venipuncture, and measurements were performed using an automated hematology analyzer (manufacturer: Sysmex Corporation, Japan; model: XN-9000) in combination with the corresponding original reagents supplied by the manufacturer to maintain standardization and analytical consistency. The analytical methodology of this instrument relies on a combination of flow cytometry and fluorescent staining techniques, specifically semiconductor laser flow cytometry, together with sheath-flow direct current (DC) detection technology, enabling precise differentiation, sizing, and enumeration of various blood cell populations. Upon completion of the automated analysis, the system generated and reported quantitative values for key CBC indices, including white blood cell count (WBC), red blood cell count (RBC), platelet count (PLT), neutrophil count (NE), lymphocyte count (LYM), along with other routine hematologic parameters, and these data were subsequently extracted from the laboratory information system for further statistical processing, monocyte count (MO), and hemoglobin (Hb). All tests were performed in accordance with standard operating procedures, and daily quality control materials were run to ensure stable instrument performance.

### Coagulation parameter testing

Coagulation indices measured within 24 h after admission were collected for all patients. In the early morning after an overnight fast, 2.7 mL of venous blood from the antecubital vein was drawn into a vacuum tube containing anticoagulant and was immediately and gently inverted 5–8 times to achieve thorough mixing of blood and anticoagulant. Within 1 h of collection, samples were centrifuged at 3,000 rpm for 15 min to separate plasma. An automated coagulation analyzer (manufacturer: Werfen, United States; model: ACL TOP 750) was used for testing. Prothrombin time (PT) and activated partial thromboplastin time (APTT) were determined by a magnetically activated clotting method, fibrinogen (FIB) concentration was measured using a clotting-based assay, and D-dimer (D-D) levels were determined by an immunoturbidimetric method. All procedures and interpretation of results were carried out strictly in accordance with the instructions provided by the instrument and reagent manufacturers.

### Blood biochemical parameter testing

For the assessment of organ function and metabolic status, all blood biochemical parameters that had been obtained within 24 h after admission were systematically retrieved and compiled for each patient in the cohort. These biochemical measurements were based on serum samples collected in the early morning under fasting conditions, during which approximately 4 mL of venous blood from the antecubital vein was drawn into a vacuum tube containing a clot activator but no anticoagulant, thereby facilitating subsequent clot formation and serum separation. After the blood samples had been allowed to stand undisturbed at room temperature for about 30 min to ensure complete coagulation, the tubes were centrifuged at 3,500 rpm for 10 min, which resulted in clear separation of the serum supernatant from the cellular components at the bottom of the tube. The isolated serum was then analyzed using an automated biochemical analyzer (manufacturer: Roche Diagnostics, Switzerland; model: Cobas c 702), and all assays were performed with the corresponding proprietary reagents and calibration materials recommended by the manufacturer to ensure methodological consistency. Specifically, C-reactive protein (CRP) concentrations were determined by an immunoturbidimetric assay, total bilirubin (TBIL) was measured using the diazo method, alanine aminotransferase (ALT) and aspartate aminotransferase (AST) activities were assessed by kinetic rate methods, albumin (ALB) levels were quantified by the bromocresol green binding technique, blood urea nitrogen (BUN) was analyzed using a urease–glutamate dehydrogenase enzymatic method, serum creatinine (Scr) was measured by the Jaffé (picric acid) reaction, and creatine kinase-MB (CK-MB) as well as lactate dehydrogenase (LDH) activities were determined by rate-based enzymatic assays. To ensure that the biochemical test results were accurate, reproducible, and stable over time, internal quality control procedures were rigorously implemented before and during each analytical run, including routine analysis of control materials and verification that all control values remained within the predefined acceptable ranges specified by the manufacturer.

### Testing of novel biomarkers

All enrolled patients had their novel biomarkers measured within 24 h of admission. These indices were mainly the mean platelet volume/platelet count ratio (MPV/PLT), the platelet-large cell ratio (P-LCR), the fibrinogen-to-albumin ratio (FAR), the fibrinogen-to-prealbumin ratio (FPR), and the coagulation-to-lymphocyte ratio (CLR). These variables are composite parameters that are mathematically derived from standard hematologic and biochemical measurements and were used to more accurately reflect platelet activation, coagulation–inflammation interactions, and nutritional–inflammatory status, and stress hyperglycemia ratio (SHR). Among these, MPV/PLT, P-LCR, FAR, FPR, and CLR were calculated as derived indices based on the aforementioned CBC, coagulation, and biochemical test results. Specifically, MPV/PLT was defined as the ratio of MPV to PLT; P-LCR represented the percentage of platelets with a volume ≥ 12 fL in the total platelet count; FAR was defined as the ratio of FIB to ALB; FPR as the ratio of FIB to prealbumin; and CLR as the ratio of FIB to LYM. Concurrently with CBC testing, fasting plasma glucose was measured using a Roche Cobas c 702 glucose analyzer and a hexokinase-based method. As part of the same laboratory work-up, glycated hemoglobin was measured concurrently with these calculations using a D-100 hemoglobin analyzer (Bio-Rad Laboratories, United States). This device operates on the principle of high-performance liquid chromatography, providing a standardized and highly reproducible measurement appropriate for the subsequent derivation of indices related to long-term glucose control. SHR was calculated as the ratio of fasting plasma glucose to glycated hemoglobin.

### Testing of novel inflammation-related indices

As part of the same laboratory work-up, glycated hemoglobin was measured concurrently with these calculations using a D-100 hemoglobin analyzer (Bio-Rad Laboratories, United States). This device operates on the principle of high-performance liquid chromatography, providing a standardized and highly reproducible measurement appropriate for the subsequent derivation of indices related to long-term glucose control. Based on above complete blood count results, NLR was defined as the ratio of NE to LYM, PLR as the ratio of PLT to LYM, and PNR as the ratio of PLT to NE. SIRI was calculated as NE × MO/LYM, and SII as PLT × NE/LYM.

### Assessment of MODS

With reference to the MODS evaluation criteria described in the *Chinese expert consensus on diagnosis and treatment of traumatic multiple organ dysfunction syndrome* (16), MODS was a clinical syndrome, 24 h after severe insult such as serious infection, trauma, shock, or major surgery, dysfunction or failure of two or more organs or systems occurs either simultaneously or sequentially. In the present study, the diagnosis of MODS was based on the internationally validated Sequential Organ Failure Assessment (SOFA) score, which enhanced the standardization of the diagnostic criteria and improved the external comparability of the study findings. MODS was defined as an increase of ≥ 2 points from the baseline SOFA score at admission in at least two organ systems during hospitalization, with the condition persisting for ≥ 48 h. All SOFA scores were independently and retrospectively evaluated by two intensivists, with an inter-rater agreement Kappa coefficient of 0.89.

### Statistical methods

Baseline data analyses were performed using SPSS version 26.0 and GraphPad Prism version 9.0. Normality of continuous variables was assessed using the Shapiro–Wilk test, while homogeneity of variance was evaluated using Levene’s test. Continuous variables with normal distribution and homogeneous variance were expressed as mean ± standard deviation; between-group comparisons were conducted using the independent-samples *t*-test, and within-group comparisons were performed using the paired-samples *t*-test. Variables that did not conform to a normal distribution were expressed as median [M (P25, P75)] and analyzed using the nonparametric Mann–Whitney U test. Categorical variables were expressed as n (%) and compared using the χ^2^ test. Among variables showing significant differences between the two groups, basic indicators involved in mathematical derivative relationships (e.g., PLT, LYM, FIB, and ALB) and composite-derived indicators (e.g., NLR, FAR, and MPV/PLT) were categorized separately, with priority given to composite indicators with stronger clinical interpretability. Multicollinearity was assessed using the variance inflation factor (VIF), and variables with VIF > 5 were excluded. Variables without collinearity issues were subsequently entered into a binary logistic regression model to identify independent influencing factors. The final model included six variables: age, time from wasp sting to hospital admission, pleural effusion, RALE score, MPV/PLT ( × 100), and SIRI. Construction and validation of the nomogram were based on the results of multivariable regression analysis. Internal validation was performed using bootstrap resampling with 1,000 iterations, and the optimism-corrected concordance index (C-index) and calibration curve were generated. Model discrimination was evaluated using the area under the receiver operating characteristic curve (AUC) and the C-index. Model calibration was visually assessed using calibration curves. Clinical net benefit was evaluated using decision curve analysis, with the threshold probability range set from 0 to 0.5. The optimal cutoff value was determined according to the maximum Youden index, and the corresponding sensitivity, specificity, positive predictive value, and negative predictive value were calculated. All statistical analyses were independently performed by two authors with expertise in biostatistics and were independently reviewed by [statistical expert name and institution to be added]. A two-sided *P* < 0.05 was considered statistically significant.

## Results

### Comparison of baseline characteristics between the MODS and NMODS groups

The median Sequential Organ Failure Assessment (SOFA) score in the MODS group at the time of diagnosis was 7 points (interquartile range: 5–9 points), indicating clinically significant organ dysfunction. Comparison of the clinical characteristics between the MODS and non-MODS (NMODS) groups demonstrated that the mean age in the MODS group was significantly higher than that in the NMODS group [(60.36 ± 12.42) years vs. (54.81 ± 12.35) years, *P* < 0.05]. The body mass index (BMI) of the MODS group was also significantly higher compared with the NMODS group [(23.88 ± 3.77) kg/m^2^ vs. (21.68 ± 3.80) kg/m^2^, *P* < 0.05]. In addition, the Visual Analogue Scale (VAS) pain score was significantly elevated in the MODS group relative to the NMODS group [(3.42 ± 1.05) vs. (3.15 ± 1.02), *P* < 0.05]. The proportions of pruritus, central necrosis, and urinary system symptoms in the MODS group were 16:29:26, respectively, which differed significantly from those in the NMODS group (73:24:3, respectively; *P* < 0.05). No statistically significant differences were observed between the two groups regarding sex, respiratory rate, heart rate, systolic blood pressure, diastolic blood pressure, sting location, number of sting sites, time from sting to hospital admission, erythema/swelling, pruritus, fever, allergic reactions, respiratory symptoms, gastrointestinal symptoms, cardiovascular symptoms, or neurological symptoms (all *P* > 0.05). It can be found in Table 1.

**TABLE 1:** Comparison of baseline characteristics between the MODS and NMODS groups.

Clinical characteristics		MODS group (*n* = 90)	NMODS group	χ^2^/*t*	*P*
Sex	Male	47	141	1.723	0.189
	Female	43	93		
Age (Years)		60.36 ± 12.42	54.81 ± 12.38	3.611	<0.001
BMI (kg/m^2^)		23.88 ± 3.77	21.68 ± 3.80	4.678	<0.001
Respiratory rate (breaths/min)		19.79 ± 4.38	19.56 ± 4.56	0.411	0.681
Heart rate (beats/min)		82.58 ± 13.74	85.20 ± 13.98	1.518	0.130
Systolic blood pressure (mmHg)		141.32 ± 15.46	138.71 ± 14.80	1.404	0.161
Diastolic blood pressure (mmHg)		83.81 ± 10.75	82.69 ± 10.62	0.855	0.393
Sting site	Head and face	39	95	0.607	0.738
	Trunk	32	80		
	Extremities	19	59		
VAS score (points)		3.42 ± 1.05	3.15 ± 1.02	2.117	0.035
Number of sting sites (n)		18.36 ± 4.42	17.96 ± 4.30	0.744	0.457
Time from sting to admission (h)		12.24 ± 3.10	12.72 ± 3.04	1.266	0.206
Erythema and swelling		87	226	0.001	0.970
Pruritus		16	73	5.875	0.015
Central necrosis		29	24	22.922	<0.001
Fever		1	4	0.125	0.911
Allergic reaction		21	57	0.037	0.847
Respiratory system symptoms		22	50	0.607	0.436
Gastrointestinal symptoms		30	62	1.495	0.221
Cardiovascular symptoms		39	91	0.534	0.465
Urinary system symptoms		26	3	60.788	<0.001
Neurological symptoms		42	104	0.130	0.719

### Comparison of chest radiograph findings between MODS and NMODS groups

Pleural effusion, cardiomegaly, pulmonary congestion, and radiographic assessment of lung edema (RALE) score were regarded as common chest radiograph indicators in patients with wasp stings, and were used to reflect the severity of pulmonary and pleural involvement caused by direct venom-induced injury and systemic inflammatory responses. When chest radiograph findings were compared between the MODS and NMODS groups, pleural effusion was observed more frequently in the MODS group than in the NMODS group, and the RALE score was also higher MODS group. While no significant differences were found for the remaining indicators (*P* > 0.05), these between-group differences were statistically significant (*P* < 0.05), showing that only specific chest radiographic findings, not all measured imaging parameters, were clearly linked to the development of MODS. These findings imply that more prominent radiographic evidence of lung involvement may serve as an early warning sign in clinical practice and that pleural effusion and RALE score may be influencing factors for the development of MODS in patients with wasp stings. The distribution of chest radiography findings, such as pleural effusion and RALE score, for both groups is shown in Table 2, which provides more details.

**TABLE 2:** Comparison of chest radiograph findings between MODS and NMODS groups.

Indicator		MODS group (*n* = 90)	NMODS group (*n* = 234)	χ^2^/*t*	*P*
Pleural effusion	Present	35	60	5.505	0.019
	Absent	55	174		
Cardiomegaly	Present	28	69	0.082	0.775
	Absent	62	165		
Pulmonary congestion	Present	20	49	0.064	0.801
	Absent	70	185		
RALE score (points)		18.36 ± 4.20	16.10 ± 4.34	4.236	<0.001

### Comparison of complete blood count indices between MODS and NMODS groups

The following routine complete blood count parameters were used to describe the patients’ systemic inflammatory and hematopoietic status: WBC, RBC, PLT, NE, LYM and associated hematologic indices, monocyte count (MO), and hemoglobin (Hb) were regarded as routine complete blood count indices and were used to assist in evaluating the baseline inflammatory response, immune status, and hematologic involvement. PLT and LYM levels were found to be lower in the MODS group than in the NMODS group when these indices were compared, while MO levels were higher in the MODS group (*P* < 0.05), suggesting that patients who experience multiple organ dysfunction may be more likely to have thrombocytopenia, lymphopenia, and monocytosis. Not all hematologic indices assessed in the CBC showed differential changes between the MODS and NMODS groups, since no statistically significant differences were found for the other parameters (*P* > 0.05). These results imply that PLT, LYM, and MO levels may be useful as readily available blood-based indicators for early risk assessment since they may be possible influencing factors for the development of MODS in patients with wasp stings. The mean values and statistical comparisons of each complete blood count parameter for the two research groups are shown in Table 3.

**TABLE 3:** Comparison of complete blood count indices between MODS and NMODS groups ($\bar{\chi }$ ± s).

Indicator	MODS group (*n* = 90)	NMODS group (*n* = 234)	*t*	*P*
WBC ( × 10^9^/L)	12.56 ± 3.74	11.96 ± 3.80	1.279	0.202
RBC ( × 10^12^/L)	4.24 ± 0.68	4.30 ± 0.59	0.785	0.433
PLT ( × 10^9^/L)	156.94 ± 42.37	191.84 ± 41.85	6.700	<0.001
NE ( × 10^9^/L)	9.82 ± 3.10	9.55 ± 3.04	0.712	0.477
LYM ( × 10^9^/L)	1.25 ± 0.34	1.54 ± 0.35	6.733	<0.001
MO ( × 10^9^/L)	0.85 ± 0.24	0.67 ± 0.22	6.430	<0.001
Hb (g/L)	128.76 ± 16.44	130.02 ± 16.98	0.604	0.524

### Comparison of coagulation indices between MODS and NMODS groups

PT, APTT, FIB, and D-D were considered the four routine coagulation indices, mainly used to evaluate the impact of wasp venom on the coagulation–fibrinolytic system and the risk of disseminated intravascular coagulation. When coagulation parameters were compared between the two groups using standard statistical tests, FIB levels were found to be markedly and significantly higher in the MODS group than in the NMODS group (*P* < 0.05), whereas the remaining coagulation indices, including the other routinely measured parameters, did not show statistically meaningful differences between groups and all had *P*> 0.05, indicating broadly similar distributions. Taken together, these results indicate that FIB levels may be specifically and independently associated with the occurrence of MODS in patients with wasp stings, potentially reflecting a closer link between hyperfibrinogenemia and the pathophysiological processes leading to multiple organ dysfunction in this setting. The detailed comparison of all coagulation indices between the MODS and NMODS groups, including the exact mean values, standard deviations, and corresponding test statistics, is presented comprehensively in Table 4.

**TABLE 4:** Comparison of coagulation indices between MODS and NMODS groups ($\bar{\chi }$ ± s).

Indicator	MODS group (*n* = 90)	NMODS group (*n* = 234)	*t*	*P*
PT (s)	12.54 ± 2.68	12.40 ± 2.52	0.440	0.660
APTT (s)	35.12 ± 4.78	34.86 ± 4.56	0.454	0.650
FIB (g/L)	5.20 ± 1.24	4.30 ± 1.10	6.363	<0.001
D-D (mg/L FEU)	1.85 ± 0.46	1.79 ± 0.42	1.121	0.263

### Comparison of blood biochemical indices between MODS and NMODS groups

CRP, TBIL, ALT, AST, ALB, BUN, Scr, CK-MB, and LDH were regarded as routine blood biochemical indices and were mainly used to assess hepatic and renal function, nutritional status, and functional impairment of myocardium and skeletal muscle, as well as other organ systems. When blood biochemical parameters were compared between the MODS and NMODS groups, In the analysis of blood biochemical parameters, ALB levels were significantly lower in the MODS group than in the NMODS group (*P* < 0.05), while all associated *P*-values were greater than 0.05. These results indicate that lower levels of ALB may be closely linked to the occurrence of MODS in wasp sting patients and may be a sign of poorer nutritional or hepatic synthetic status that predisposes to multiple organ dysfunction, while other routine biochemical markers appear less discriminative in this context. The detailed results for each biochemical parameter, including ALB and the remaining indices, are shown in Table 5, which also displays the distributions and comparative statistics for both study groups.

**TABLE 5:** Comparison of blood biochemical indices between MODS and NMODS groups ($\bar{\chi }$ ± s).

Indicator	MODS group (*n* = 90)	NMODS group (*n* = 234)	*t*	*P*
CRP (mg/L)	48.45 ± 12.23	46.98 ± 12.45	0.957	0.340
TBIL (μmol/L)	18.57 ± 6.10	17.84 ± 5.92	0.986	0.325
ALT (U/L)	45.32 ± 12.24	43.98 ± 12.35	0.877	0.381
AST (U/L)	48.76 ± 15.56	46.22 ± 15.40	1.326	0.186
ALB (g/L)	32.62 ± 5.84	36.90 ± 4.97	6.604	<0.001
BUN (g/L)	7.24 ± 2.62	6.99 ± 2.53	0.789	0.431
Scr (μmol/L)	98.64 ± 22.35	96.80 ± 21.87	0.674	0.501
CK-MB (U/L)	27.86 ± 5.42	26.90 ± 5.35	1.441	0.150
LDH (U/L)	320.45 ± 56.45	316.98 ± 55.24	0.503	0.615

### Comparison of novel biomarker levels between MODS and NMODS groups

MPV/PLT, P-LCR, FAR, FPR, CLR, and SHR were considered novel biomarkers, reflecting key pathophysiological processes such as the inflammation–coagulation cascade, platelet activation, and stress-related metabolic derangements. When these novel biomarkers were compared between the two groups, the MODS group consistently had higher levels of MPV/PLT, P-LCR, FAR, FPR, CLR, and SHR than the NMODS group. All of these differences were statistically significant with *P*-values less than 0.05. These findings suggest that the aforementioned novel biomarkers may be closely linked to the development of MODS in wasp sting patients, and they collectively imply that changes in coagulation-inflammation ratios, platelet activation indices, and stress-related glycemic markers are more noticeable in those who progress to multiple organ dysfunction. The specific data, including mean values, variability measures, and statistical comparisons for each of these composite biomarkers in the MODS and NMODS groups are detailed in Table 6.

**TABLE 6:** Comparison of novel biomarker levels between MODS and NMODS groups ($\bar{\chi }$ ± s).

Indicator	MODS group (*n* = 90)	NMODS group (*n* = 234)	*t*	*P*
MPV/PLT	0.06 ± 0.02	0.05 ± 0.01	5.961	<0.001
P-LCR	32.54 ± 8.46	25.98 ± 7.94	6.540	<0.001
FAR	0.16 ± 0.04	0.13 ± 0.04	6.047	<0.001
FPR	0.19 ± 0.05	0.15 ± 0.05	6.450	<0.001
CLR	4.32 ± 1.11	3.65 ± 0.74	6.293	<0.001
SHR	1.28 ± 0.34	1.02 ± 0.30	6.728	<0.001

### Comparison of inflammation-related indices between MODS and NMODS groups

NLR, PLR, PNR, SIRI, and SII were considered routine inflammation-related indices, and were used to quantify the severity of systemic inflammatory response and the status of immune balance. When inflammation-related indices were systematically compared between the MODS and NMODS groups, levels of NLR, PLR, PNR, SIRI, and SII in the MODS group were found to be significantly higher than those in the NMODS group (P < 0.05 for all comparisons), indicating a more intense systemic inflammatory and immune response among patients who developed MODS. These findings suggest that these inflammation-related indices may act as convenient and integrative blood-based markers for identifying people who are at increased risk of multiple organ dysfunction. Table 7 provides comprehensive reference.

**TABLE 7:** Comparison of inflammation-related indices between MODS and NMODS groups ($\bar{\chi }$ ± s).

Indicator	MODS group (*n* = 90)	NMODS group (*n* = 234)	*t*	*P*
NLR	9.16 ± 2.24	7.38 ± 2.10	6.707	<0.001
PLR	134.64 ± 35.28	104.78 ± 34.80	6.891	<0.001
PNR	16.85 ± 3.84	20.04 ± 4.20	6.267	<0.001
SIRI	6.26 ± 1.40	4.98 ± 1.35	7.566	<0.001
SII	1250.48 ± 220.86	1075.62 ± 210.78	6.600	<0.001

### Factors influencing the development of MODS in patients with wasp stings

To avoid multicollinearity, variables with statistical significance in the univariate analysis were categorized into basic indicators (e.g., PLT, LYM, MO, FIB, and ALB) and their derived composite indicators (e.g., NLR, PLR, PNR, SII, SIRI, FAR, FPR, CLR, MPV/PLT, and P-LCR). Considering that composite-derived indicators can more comprehensively reflect the intrinsic interactions among multiple pathophysiological pathways, including inflammation and coagulation, and possess greater clinical interpretability, these composite indicators were preferentially incorporated into the initial logistic regression model. Variables exhibiting collinearity between basic indicators and their corresponding derived indicators (VIF > 5) were excluded. The final model included six variables: age, time from wasp sting to hospital admission, pleural effusion, RALE score, MPV/PLT ( × 100), and SIRI. Binary logistic regression analysis demonstrated that RALE score (OR = 1.145, 95% CI: 1.068–1.232, *P* < 0.001), MPV/PLT × 100 (OR = 1.544, 95% CI: 1.279–1.893, *P* < 0.001), and SIRI (OR = 1.928, 95% CI: 1.562–2.430, *P* < 0.001) were independent risk factors for the development of MODS in patients with wasp stings. Although age, time from sting to hospital admission, and pleural effusion did not reach statistical significance in the multivariable analysis (*P* > 0.05), these variables were retained in the nomogram due to their clinical relevance and *P*-values approaching 0.05, allowing for comprehensive risk assessment (Table 8).

**TABLE 8:** Binary logistic regression analysis of factors associated with MODS in patients with wasp stings.

Indicator	B coefficient	SE	Wald *X*^2^	*P*	OR	95%CI
Age	0.016	0.012	1.885	0.170	1.016	0.994∼1.040
Time from sting to admission	0.085	0.049	2.966	0.085	1.089	0.989∼1.201
Pleural effusion	0.572	0.316	3.283	0.070	1.771	0.952∼3.292
RALE score	0.135	0.036	13.838	<0.001	1.145	1.068∼1.232
MPV/PLT	0.435	0.100	19.025	<0.001	1.544	1.297∼1.893
SIRI	0.656	0.112	34.132	<0.001	1.928	1.562∼2.430

### Construction of a nomogram-based risk prediction model for MODS in patients with wasp stings

**FIGURE 1:**
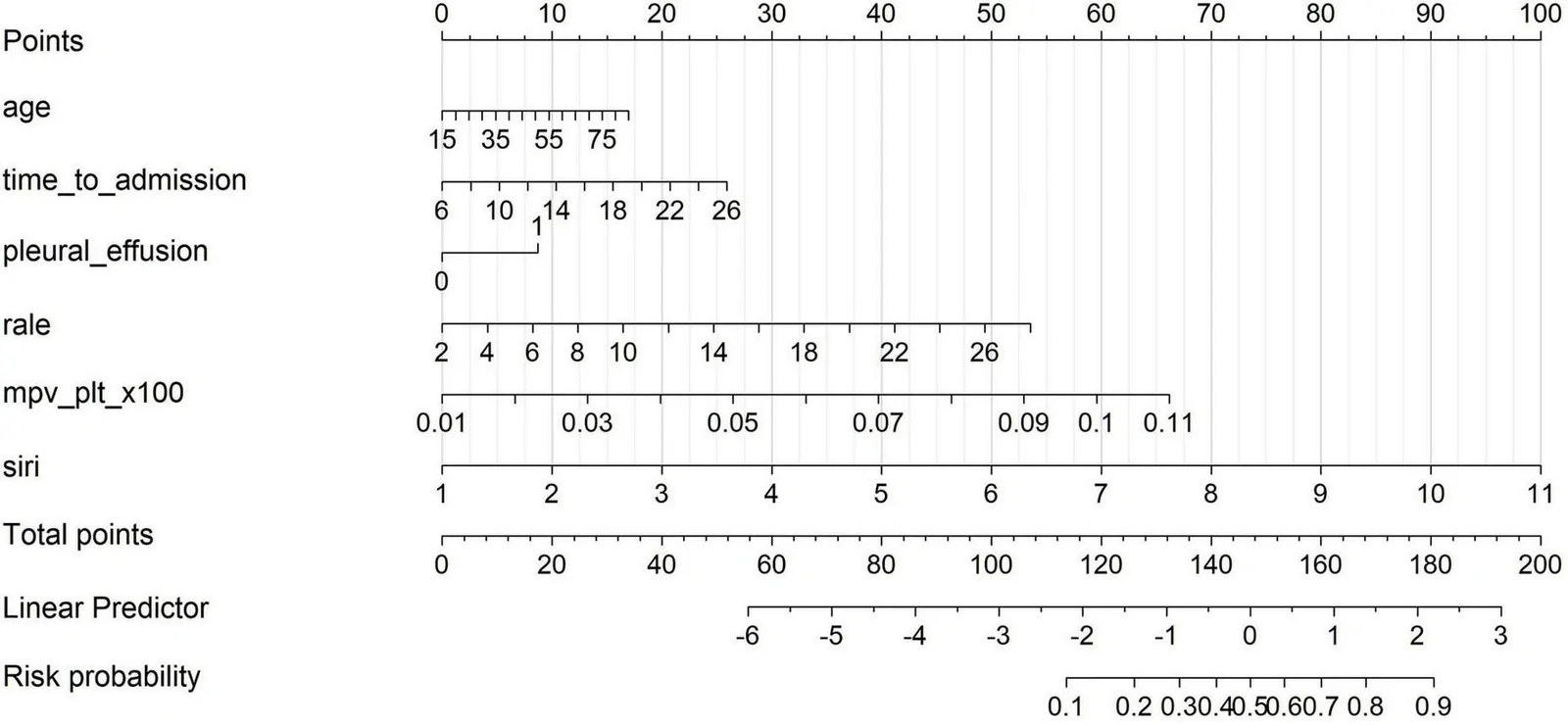
Nomogram-based risk prediction model for the occurrence of MODS in patients with wasp stings.

Based on the results of the aforementioned binary logistic regression analysis, the following nomogram model was established: Logit(P) = −11.767 + 0.016 × age + 0.085 × time from wasp sting to hospital admission + 0.572 × pleural effusion (yes = 1) + 0.135 × RALE score + 0.435 × (MPV/PLT × 100) + 0.656 × SIRI. Based on the six selected predictive factors, namely age, time from wasp sting to hospital admission, pleural effusion, RALE score, MPV/PLT, and SIRI, a visualized scoring system was constructed. Each risk factor corresponded to an independent scale axis, and the length of each scale intuitively reflected the relative contribution weight of that factor to prognosis. The total score was calculated by summing the points assigned to each variable, and the individualized risk probability was subsequently mapped onto the probability scale, thereby enabling quantitative assessment of the risk of MODS occurrence in patients with wasp stings (see Figure 1).

### Performance evaluation of the nomogram-based risk prediction model for MODS in patients with wasp stings

**FIGURE 2:**
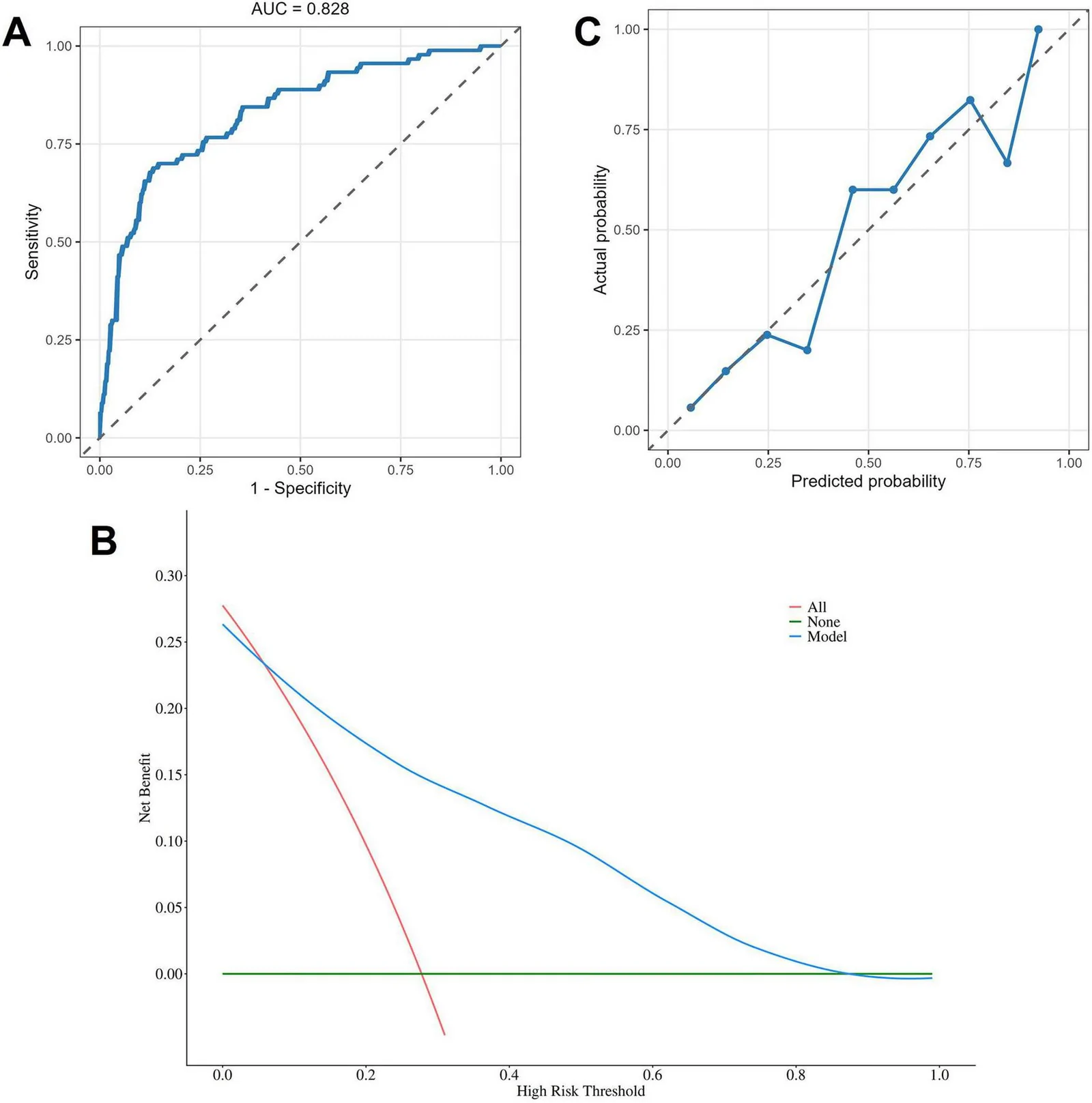
**(A)** The ROC curve. **(B)** Decision curve analysis. **(C)** Calibration curve.

Receiver operating characteristic (ROC) curve analysis demonstrated that the combined prediction model achieved an AUC of 0.828 (95% CI: 0.776–0.880), as shown in Figure 2A. Using the prediction probability corresponding to the maximum Youden index (0.368) as the cutoff value, the model yielded a sensitivity of 66.7%, specificity of 87.9%, positive predictive value of 68.9%, and negative predictive value of 86.8% for predicting MODS. Internal validation using 1,000 bootstrap resamples showed an optimism-corrected C-index of 0.811 and a Nagelkerke R^2^ of 0.383. The decision curve analysis demonstrated a high net clinical benefit (Figure 2B), while the calibration curve closely approximated the ideal reference line (Figure 2C), indicating good calibration performance.

## Discussion

As a clinical emergency, wasp sting injury is serious not only because of its local toxic effects, but more importantly because of the potentially fatal systemic inflammatory response and MODS that may be triggered (17, 18). MODS represents the central link leading to death in patients with wasp stings; essentially, it is a cascading, avalanche-like process of uncontrolled systemic inflammation and immune–metabolic dysregulation initiated by venom toxins. However, in current practice, effective tools capable of identifying high-risk patients early and accurately are still lacking (19, 20). From the above perspectives, the present study systematically identified RALE score, MPV/PLT, and SIRI as independent risk factors for MODS. Although age, time from wasp sting to hospital admission, and pleural effusion showed significant intergroup differences in the univariate analysis, they did not reach statistical significance in the multivariable regression analysis. Considering their potential clinical relevance, these variables were retained in the prediction model. An individualized risk prediction model with favorable clinical utility was subsequently developed and validated, which may facilitate early identification of high-risk patients and support targeted clinical interventions.

Results this study showed that patients in the MODS were older than those NMODS, and time from sting to hospital admission was longer MODS than in the NMODS group; both variables were identified as independent risk factors for MODS in patients with wasp stings. Increasing age generally indicates a decline in physiological organ reserve and a reduced ability to cope with intense stress, which makes elderly patients more prone to functional decompensation after a wasp sting (21). Once the sting has occurred, a prolonged interval before admission inevitably leads to a longer duration of toxin action in the body and delays the critical therapeutic window during which medical interventions can remove venom and interrupt the inflammatory cascade, thereby increasing the risk of MODS (22). Clinically, close attention should therefore be paid to older patients with wasp stings, and they should be advised to seek medical care promptly after onset in order to prevent further deterioration and the development of severe complications such as MODS.

With regard to chest radiography, pleural effusion and abnormal RALE scores often carry clear pathophysiological implications. Wasp venom and the ensuing systemic inflammatory response can directly injure pulmonary capillary endothelial cells and alveolar epithelial cells, increase vascular permeability, and consequently lead to pulmonary edema and pleural effusion (23, 24). As a semi-quantitative imaging tool, the RALE score permits accurate quantification of the extent and severity of such lung injury (25). The present study found that pleural effusion was more common in the MODS group than in the NMODS group, and the RALE score was significantly higher in the MODS group. These findings suggest that abnormalities on chest radiography are not only manifestations of respiratory system involvement, but also a direct reflection of systemic injury induced by wasp venom in the lungs as a target organ, potentially indicating the initiation of the MODS process. Therefore, close monitoring of chest radiographic findings after admission is warranted, and individualized prevention and treatment strategies should be formulated based on imaging results.

Higher MPV/PLT and SIRI levels were found in the MODS group compared to the NMODS group in this study. These two indices were found to be independent risk factors for MODS in wasp sting patients, indicating that increased systemic inflammatory response and platelet activation are important factors in the pathophysiology of venom-induced multiple organ dysfunction. Components of wasp venom, such as hemolytic peptides, can disrupt phospholipid structures in platelet membranes, leading to increased platelet destruction and coagulation dysfunction. At the same time, hyaluronidase and phospholipase A2 in the venom can aggravate vascular endothelial injury, further enhancing platelet consumption and coagulation abnormalities (26, 27). Variations in MPV/PLT reflect the degree of platelet activation and the status of coagulation function; marked increases or decreases in this index indicate a higher risk of MODS (28). In addition, venom-induced systemic inflammation promotes the release of inflammatory cytokines and activation of the systemic inflammatory response. As an integrated inflammatory marker, SIRI tends to rise in parallel with the risk and severity of MODS (29, 30). In this context, dynamic monitoring of MPV/PLT and SIRI in patients with wasp stings should be strengthened in clinical practice. Timely anti-inflammatory therapy, correction of coagulation abnormalities, and, when necessary, organ support therapy should be implemented to preserve organ function and suppress the occurrence and progression of MODS.

The main innovation is construction nomogram-based risk prediction model derived from binary Logistic regression analysis. This model integrates baseline clinical variables (age, time from sting to admission), imaging indices (pleural effusion, RALE score), and laboratory parameters (MPV/PLT, SIRI) as predictors. The resulting nomogram provides a visual and easily interpretable tool for estimating the probability of MODS patients of wasp stings. The ROC curve demonstrated a relatively high AUC, the calibration curve closely approximated the reference line, and the decision curve remained above the two extreme curves, together showing model exhibits nice discrimination, calibration, as well as net clinical benefit. These results confirm that the nomogram has meaningful clinical utility for predicting MODS after wasp stings. It should be noted that, although great varieties of MODS and NMODS were researched PLT, LYM, ALB, MO, FIB, P-LCR, FAR, FPR, CLR, SHR, NLR, PLR, PNR, and SII, these indices were not retained as independent influencing factors in the binary Logistic regression analysis. Taken together, the independent risk factors identified in this model collectively delineate the clinical profile of patients at high risk for developing MODS following wasp stings, characterized by advanced age, delayed hospital presentation, a coagulation–platelet activation state reflected by elevated MPV/PLT, marked systemic inflammation indicated by increased SIRI, and acute pulmonary injury manifested by pleural effusion and elevated RALE scores. This pattern suggests that uncontrolled interaction between inflammatory and coagulation networks, with the lungs serving as an early target organ, represents a central pathophysiological pathway driving the progression of MODS after wasp stings.

This study has several limitations. First, only 324 patients with wasp stings from a single center were included, and no post-discharge follow-up was performed. This introduces selection bias, limits generalizability, and prevents the acquisition of long-term outcome data. Second, the retrospective design may be associated with incomplete or missing records, potentially affecting the comprehensiveness of variable selection and the accuracy of result interpretation. The most significant limitation of the present study is the absence of an independent external validation cohort, which fundamentally restricts the generalizability of the model to other regions, healthcare settings, and patient populations. Before this nomogram can be recommended for routine clinical practice, rigorous external validation through prospective, multicenter studies is essential. In addition, the relatively limited sample size and the lack of statistical significance of variables such as age and time to hospital admission in the multivariable analysis may be attributable to sample size limitations and collinearity. Future studies with larger sample sizes are therefore warranted for further validation. In light of these limitations, future research should adopt multicenter, large-sample prospective designs to improve sample representativeness. Independent validation cohorts should be obtained through multi-institutional collaboration to confirm the clinical applicability and external validity of the model. By doing so, the objectivity, comprehensiveness, and scientific robustness of the findings may be enhanced, thereby providing more reliable and substantial support for clinical decision-making in the management of patients with wasp stings.

### Conclusion

In summary, the independent risk factors for MODS in patients with wasp stings mainly included RALE score, MPV/PLT, and SIRI levels, whereas the independent effects of age, pleural effusion, and time to hospital admission were not confirmed. The nomogram-based risk prediction model established accordingly still demonstrated favorable discrimination and clinical utility. However, the model lacks external validation, and the conclusions require further confirmation through future large-scale, multicenter, and prospective longitudinal studies.

## Data Availability

The original contributions presented in this study are included in the article/supplementary material, further inquiries can be directed to the corresponding authors.
