# Correction: Prediction model for risk of multiple organ dysfunction syndrome in wasp sting patients: development and validation

**DOI:** 10.3389/fmed.2026.1922779

**Published:** 2026-10-26

**Authors:** Hongmei Dai, Dongmei Liu, Li Yang, Jiawei Chen, Xiaogang Du

**Affiliations:** 1 Health Management Center, The First Affiliated Hospital of Chongqing Medical University, Chongqing, China; 2 Department of Nephrology and Hematology, Yunyang County People’s Hospital, Chongqing, Yunyang County, China; 3 Metabolism and Immunology Laboratory for Urological Diseases, The First Affiliated Hospital of Chongqing Medical University, Chongqing, China

**Keywords:** associated factors, multiple organ dysfunction syndrome, nomogram, risk prediction model, wasp sting

Affiliation ‘Health Management Center, The First Affiliated Hospital of Chongqing Medical University, Chongqing 400016, China’ was omitted from the paper. This affiliation has now been added for authors Hongmei Dai and Xiaogang Du.

Affiliation ‘Metabolism and Immunology Laboratory for Urological Diseases, The First Affiliated Hospital of Chongqing Medical University, Chongqing 400016, China’ was erroneously given as ‘Department of Nephrology, The First Affiliated Hospital of Chongqing Medical University, Chongqing 400016, China’.

The original version of this article has been updated.

